# β-Thujaplicin induces autophagic cell death, apoptosis, and cell cycle arrest through ROS-mediated Akt and p38/ERK MAPK signaling in human hepatocellular carcinoma

**DOI:** 10.1038/s41419-019-1492-6

**Published:** 2019-03-15

**Authors:** Guangya Zhang, Jiangping He, Xiaofei Ye, Jing Zhu, Xi Hu, Minyan Shen, Yuru Ma, Ziming Mao, Huaidong Song, Fengling Chen

**Affiliations:** 1 Department of Endocrinology, Shanghai Ninth People’s Hospital, Shanghai Jiao Tong University School of Medicine, Shanghai, 201999 China; 2 Department of Endocrinology, Affiliated Hangzhou First People’s Hospital, Zhejiang University School of Medicine, Hangzhou, 310022 China; 3 School of Public Health, Shanghai Jiao Tong University School of Medicine, Shanghai, 200025 China; 4 Bengbu Medical College, Bengbu, 233040 China; 5 The Core Laboratory in Medical Center of Clinical Research, Shanghai Ninth People’s Hospital, Shanghai Jiao Tong University School of Medicine, Shanghai, 200025 China

## Abstract

Hepatocellular carcinoma (HCC), a common liver malignancy worldwide, has high morbidity and mortality. β-Thujaplicin, a tropolone derivative, has been used in some health-care products and clinical adjuvant drugs, but its use for HCC is unknown. In this study, we found that β-Thujaplicin inhibits the growth of HCC cells, but not normal liver cells, with nanomolar potency. Mechanistically, we found that β-Thujaplicin could induce autophagy, as judged by western blot, confocal microscopy, and transmission electron microscopy. Further using β-Thujaplicin combined with an autophagy blocker or agonist treatment HepG2 cells, we found that β-Thujaplicin induced autophagic cell death (ACD) mediated by ROS caused inhibition of the Akt-mTOR signaling pathway. Moreover, β-Thujaplicin triggered HepG2 apoptosis and increased cleaved PARP1, cleaved caspase-3, and Bax/Bcl-2 ratio, which indicated that β-Thujaplicin induced apoptosis mediated by the mitochondrial-dependent pathway. We also found that increased expression of p21 and decreased expression of CDK7, Cyclin D1, and Cyclin A2 participating in β-Thujaplicin caused the S-phase arrest. It seems that β-Thujaplicin exerts these functions by ROS-mediated p38/ERK MAPK but not by JNK signaling pathway activation. Consistent with in vitro findings, our in vivo study verified that β-Thujaplicin treatment significantly reduced HepG2 tumor xenograft growth. Taken together these findings suggest that β-Thujaplicin have an ability of anti-HCC cells and may conducively promote the development of novel anti-cancer agents.

## Introduction

Hepatocellular carcinoma (HCC) is the most common primary liver cancer and the sixth most frequent neoplasm^1^. Despite the fact that the diagnosis and treatment of HCC have been advanced, most HCC patients present an unresectable tumor and a limited choice of treatment at diagnosis^2^. Recently, two multikinase inhibitors, sorafenib and lenvatinib, have confirmed delays tumor progression in advanced HCC, which have been used as a selective method to treat advanced HCC^3,4^. However, a recent phase 3 non-inferiority trial revealed that using lenvatinib or sorafenib as a first-line treatment for unresectable HCC, the median survival time was only 13.6 and 12.3 months, respectively^5^. Therefore, it is imperative to develop novel effective anti-HCC drugs to minimize the mortality of HCC patients.

β-Thujaplicin, a natural tropolone derivative, has been identified to exhibit a variety of biological properties, including antibacterial, antifungal, antiviral, anti-inflammatory, and anticancer potential^6–13^. β-Thujaplicin has been used in some health-care products, such as cosmetics, toothpastes, and body soaps^14^. Recent data suggested that β-Thujaplicin inhibited tumor formation of human colon cancer cells through the S-phase arrest and DNA demethylation^6,8^. Although it was reported that β-Thujaplicin inhibited few types of cancer cell growth, its antitumor activity and mechanisms on HCC cells have not been investigated.

Autophagy is a highly conserved cellular self-digestion process in which cellular long-lived proteins or organelles are sequestered into the autolysosomes to be degraded or recycled. It can be triggered by a variety of stimuli, such as nutrient deprivation, protein aggregates, and reactive oxygen species (ROS)^15^. Normally, autophagy is a cellular quality control and stress response mechanism in a pro-survival manner. However, there is an increasing evidence for autophagy-related cell death, in particular in autophagic cell death (ACD), which is also known as type II programmed cell death^16–18^. Among the numerous molecular mechanisms involved in regulating autophagy, serine/threonine-protein kinases (Akt) and mammalian targets of rapamycin (mTOR) constitute the most pivotal node of the signaling pathway. The activated Akt-mTOR delays the death of cancer cells and promotes their proliferation^15^. Therefore, targeting this pathway may result in autophagic cancer cell death, and could be used for antitumor treatment.

In addition to ACD, apoptosis, also known as type I programmed cell death, is considered to be the major method of eradicating cancers^19^. Recent evidence indicates that some proteins involved in antagonizing apoptosis, such as Bcl-XL, XIAP, and Mcl-1, are overexpressed in HCC. Meanwhile, some proteins that exert a survival function, such as p53, Bcl-2, and vascular endothelial growth factor, are upregulated in HCC^20,21^. The expression and/or activation of the pro-survival RAS/ERKs and PI3K-Akt pathways are upregulated in many HCC cells^20^. Interestingly, the antitumor effect of sorafenib is also achieved by promoting HCC cell apoptosis^3^. Thus, other drugs that improve apoptosis sensitivity represent an attractive therapeutic strategy for cancer therapy.

In the present study, we demonstrated that β-Thujaplicin is effective against HCC cells in vitro and in vivo. Our observations showed that β-Thujaplicin effectively inhibits HCC cells proliferation, but is minimally toxic to normal liver cells. Mechanistically, we found that ACD, apoptosis, and S-phase arrest are involved in the effect of β-Thujaplicin in HCC cells. Moreover, our data revealed that the cytotoxicity of β-Thujaplicin is closely associated with the suppression of the Akt-mTOR and activation of p38/ERK MAPK pathways, which were dependent on the accumulation of ROS. Our results validate β-Thujaplicin as a potential therapeutic agent for HCC cancer.

## Results

### β-Thujaplicin inhibits viability and colony formation of human HCC cells

**Fig. 1:**
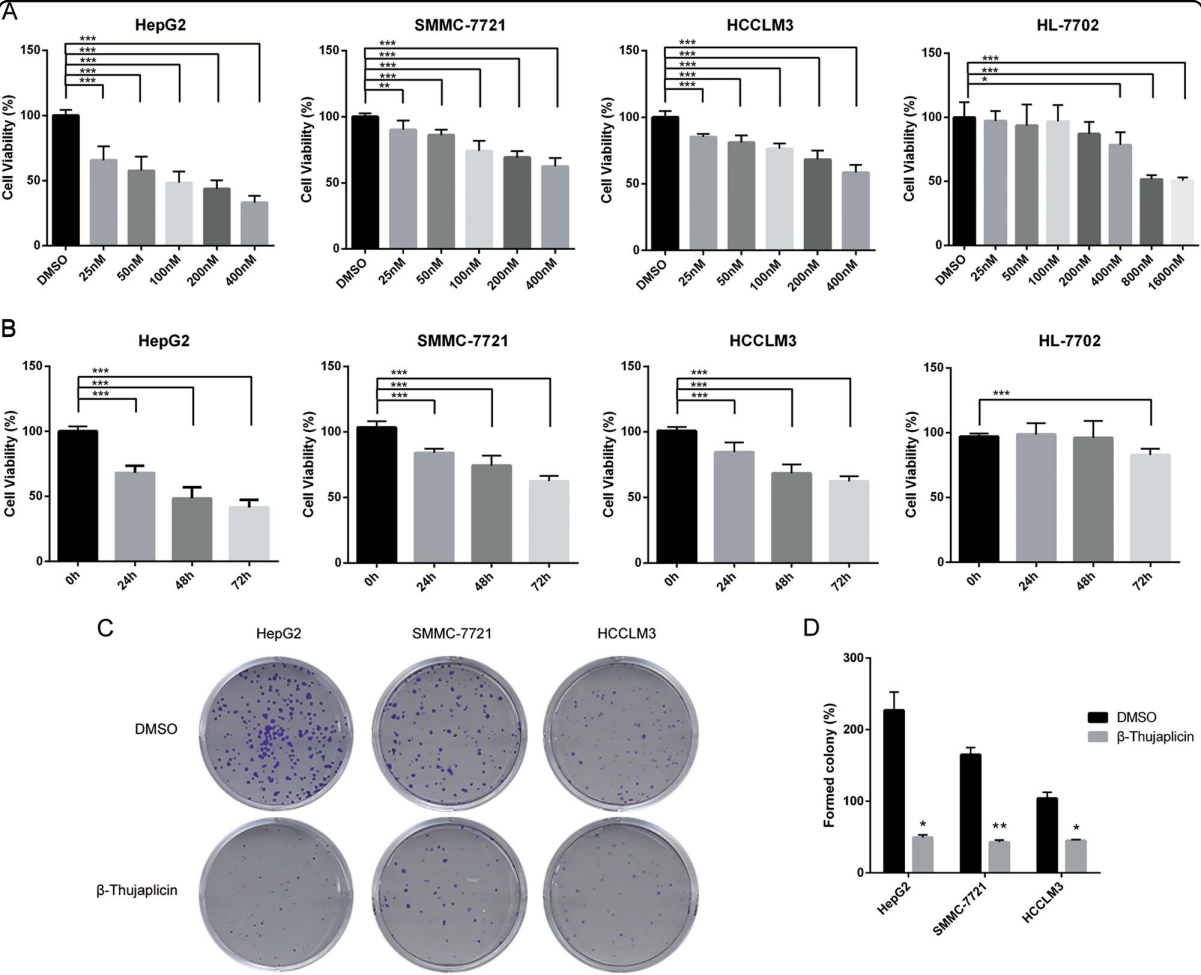
β-Thujaplicin inhibits human hepatocellular carcinoma (HCC) cell growth and colony formation. **a** HCC cell lines HepG2, SMMC-7721, HCCLM3, and normal liver cell line HL-7702 cells were treated with different concentrations of β-Thujaplicin or 0.2% dimethyl sulfoxide (DMSO) for 24 h, and the 3-(4, 5-dimethylthiazol-2-yl)−2, 5- diphenyltetrazolium bromide (MTT) assay was used to measure cell viability. **b** HepG2, SMMC-7721, HCCLM3, and HL-7702 cells were treated with 100 nM β-Thujaplicin or 0.2% DMSO for 24, 48, and 72 h, and cell proliferation was measured with a MTT assay. **c** Clone formation assay of HepG2, SMMC-7721, and HCCLM3 cells with 0.2% DMSO or 100 nM β-Thujaplicin. **d** Quantification of the mean number of formed clones in each group. All experiments were performed in triplicate. β-Thujaplicin-treated vs. DMSO-treated; *P < 0.05, **P < 0.01, ***P < 0.001

To evaluate the cytotoxicity of β-Thujaplicin, we treated HCC cell lines (HepG2, SMMC-7721, and HCCLM3) and normal liver cell line HL-7702 with different concentrations of β-Thujaplicin (25, 50, 100, 200, and 400 nM, dimethyl sulfoxide (DMSO) was used as a control) for 24, 48, and 72 h. Then 3-(4, 5-dimethylthiazol-2-yl)−2,5-diphenyltetrazolium bromide (MTT) assays were used to analyze cell viability. As shown in Fig. 1a, HepG2, SMMC-7721, and HCCLM3 cells treated with β-Thujaplicin showed a sharp decline in the cell viability in a dose-dependent manner compared with the DMSO group. Interestingly, there was only a slight decrease in cell viability when the β-Thujaplicin concentration was 400 nM in HL-7702 (Fig. 1a). In addition, levels of autophagy and apoptosis and distribution of the cell cycle in β-Thujaplicin-treated HL-7702 cells were similar to controls (Fig. S2). These results suggest that β-Thujaplicin was more cytotoxic in cancer cells compared with normal liver cells. Actually, 100 nM β-Thujaplicin can significantly inhibit the growth of HCC cells and have similar effects with higher concentrations. So, we chose 100 nM β-Thujaplicin for further investigation. Further treatment with β-Thujaplicin at 100 nM for 24, 48, and 72 h resulted in an increasing inhibition of HCC cell viability (Fig. 1b), but only has a trifling inhibition on HL-7702 at 72 h. The inhibitory effect of β-Thujaplicin on HCC cells was confirmed by a colony formation assay. As shown in Fig. 1c, d, β-Thujaplicin decreased the formation of HCC cell colonies in a dose-dependent manner.

### β-Thujaplicin induces ACD mediated by the Akt-mTOR pathway in HepG2 cells

**Fig. 2:**
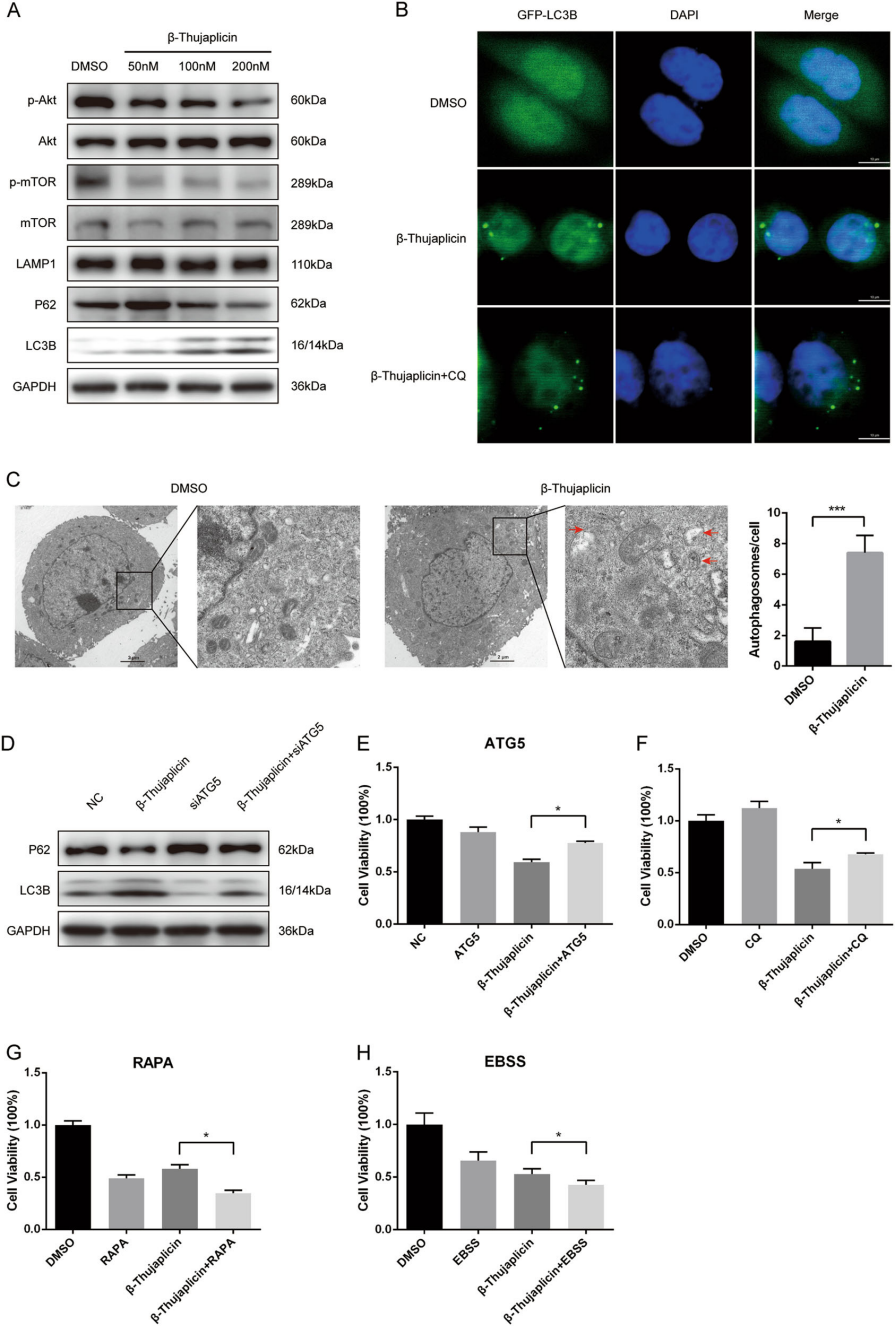
β-Thujaplicin induces autophagic cell death in HepG2 cells. **a** HepG2 cells were treated with increasing concentrations of β-Thujaplicin or 0.2% dimethyl sulfoxide (DMSO) for 24 h, and then the levels of autophagy-related proteins (p-Akt, Akt, p-mTOR, mTOR, LAMP1, p62, and LC3B) were determined by western blot. **b** HepG2 cells were infected with Ad-GFP-LC3 adenovirus for 24 h and pretreated with vehicle or 50 μM chloroquine (CQ) for 6 h, and then exposed to 100 nM β-Thujaplicin or 0.2% DMSO for another 24 h. GFP-LC3 punctae were acquired by confocal microscopy. **c** Representative transmission electron microscopy pictures of autophagosomes (arrow) in β-Thujaplicin or the DMSO group. **d** Immunoblot analysis of p62 and LC3B of cell lysates derived from control or ATG5 knockdown HepG2 cells treated with 100 nM β-Thujaplicin or 0.2% DMSO. **e**, **f** The cytotoxic activity of β-Thujaplicin can be attenuated by small interfering RNA against ATG5 and pretreatment with an autophagy inhibitor CQ. **g**, **h** β-Thujaplicin-induced cell death can be aggravated by autophagy activators Earle’s balanced salt solution (EBSS) and rapamycin (RAPA)

To determine whether β-Thujaplicin treatment resulted in autophagy, autophagosome formation and autophagy flux were investigated. Western blot results showed that phosphorylation of Akt and mTOR was decreased and the expression of LAMP1 and LC3B-II was significantly increased with β-Thujaplicin treatment in a dose-dependent manner, suggesting that autophagosome formation was induced. Interestingly, with the concentration of β-Thujaplicin increasing, the protein levels of p62, a selective receptor of autophagy substrates, were increased first and then decreased (Fig. 2a). To further confirm the induction of autophagosomes, we performed fluorescence imaging of GFP-LC3, in which the punctate staining of GFP-LC3 symbolizes the autophagosomes. As shown in Fig. 2b, β-Thujaplicin treatment significantly increased GFP-LC3 dots compared with DMSO treatment, both in the presence and absence of chloroquine (CQ), which blocks autophagy flux by inhibiting the fusion of autophagosomes and lysosomes, demonstrating that autophagy flux was indeed enhanced in HepG2 cells. To observe the autophagosomes or autolysosomes more directly, we used transmission electron microscopy and found that HepG2 cells formed more autophagic vacuoles under β-Thujaplicin treatment than the DMSO group (Fig. 2c). These results suggest that β-Thujaplicin induces autophagy in HCC cells.

To verify whether autophagy is responsible for β-Thujaplicin-induced cell death, we silenced Atg5, an E3 ubiquitin-like ligase necessary for autophagy by small interfering RNA (siRNA) (Fig. S1a, b). HepG2 cells with ATG5 knockdown inhibited β-Thujaplicin-induced increase of LC3B-II/I and decrease of P62 (Fig. 2d) and rescued β-Thujaplicin-induced cell death (Fig. 2e). It suggested that stimulated autophagy is involved in β-Thujaplicin-induced cell death. Moreover, inhibition of the autophagic flux with CQ significantly blocked the β-Thujaplicin-induced reduction of cell viability (Fig. 2f). However, treatment with rapamycin (RAPA) and Earle’s balanced salt solution (EBSS), two autophagy activators, increased β-Thujaplicin-induced cell death (Fig. 2g, h). Taken together, these results indicated that autophagy plays a role in the β-Thujaplicin-promoted HCC cell death.

### β-Thujaplicin induces apoptosis of HepG2 cells in an intrinsic pathway

**Fig. 3:**
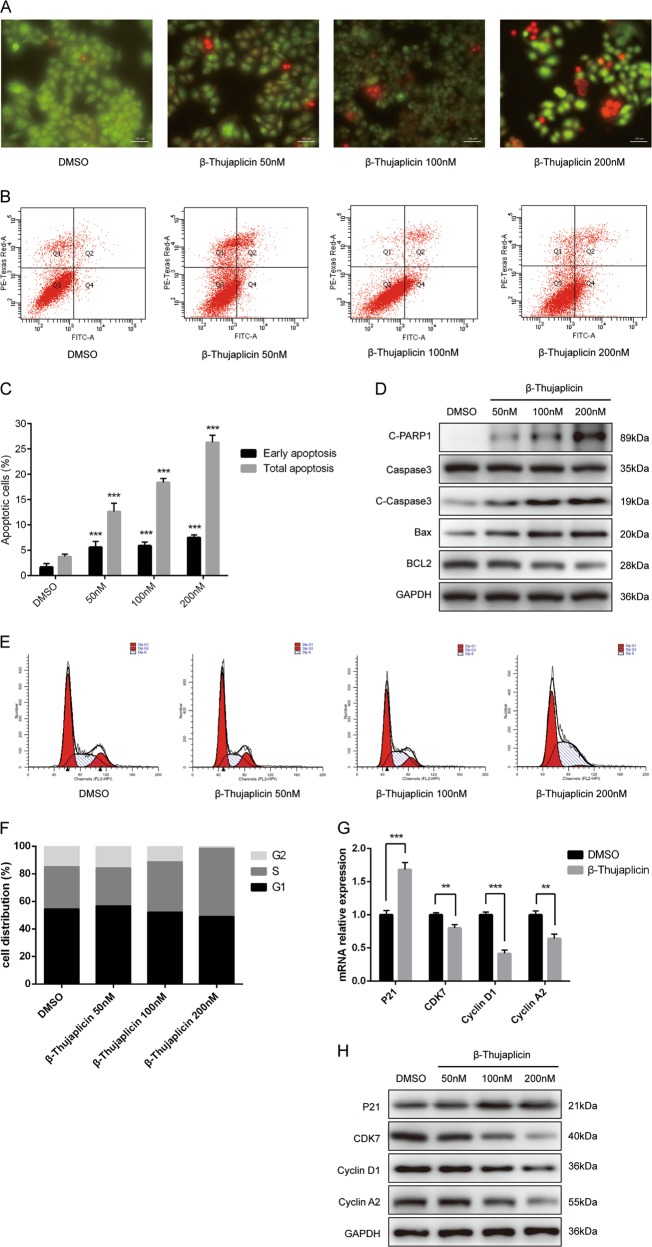
β-Thujaplicin induces apoptosis and S-phase arrest of HepG2 cells. **a** Treatment of HepG2 cells with increasing concentrations of β-Thujaplicin or 0.2% dimethyl sulfoxide (DMSO) for 24 h and the apoptotic cells were detected using acridine orange/ethidium bromide staining. Cells exhibiting perinuclear bright green patches and orange to red nucleic fluorescence indicated early and late apoptosis. **b**, **c** HepG2 cells were treated with different concentrations of β-Thujaplicin for 24 h, and then the ratio of apoptosis was measured and analyzed by Annexin V-FITC/PI staining and flow cytometry. **d** HepG2 cells were treated with 50, 100, and 200 nM of β-Thujaplicin or 0.2% DMSO for 24 h, and then the levels of apoptosis-related proteins (c-PARP1/PARP1, c-Caspase-3/Caspase-3, Bax, and Bcl-2) were determined by western blot. **e**, **f** HepG2 cells treated with β-Thujaplicin (50, 100, and 200 nM) or 0.2% DMSO were stained with PI and flow cytometry was used to analyze the cell cycle distribution. **g** Relative mRNA expression of the S-phase arrest-related genes (*P21*, *CDK7*, *Cyclin D1*, and *Cyclin A2*) was measured after 100 nM β-Thujaplicin treated for 24 h. **h** HepG2 cells were treated with the indicated concentrations of β-Thujaplicin or 0.2% DMSO for 24 h. The levels of the S-phase arrest-related proteins (P21, CDK7, Cyclin D1, and Cyclin A2) were determined by western blot. All experiments were performed in triplicate. β-Thujaplicin-treated vs. DMSO-treated; **P < 0.01, ***P < 0.001

To figure out if apoptosis participates in the inhibition of cell growth triggered by β-Thujaplicin, we performed acridine orange/ethidium bromide (AO/EB) staining. Compared with the control group, β-Thujaplicin treatment group cells stained with more perinuclear bright green patches and orange to red nuclei, indicated that β-Thujaplicin increases both early and late apoptotic cells (Fig. 3a). Flow cytometric analysis data also confirmed that both early and late apoptosis were significantly increased. As shown in Fig. 3b, c, the HepG2 cell treated with doses of 50, 100, and 200 nM β-Thujaplicin, increased the apoptotic rates to 12.63 ± 1.65%, 18.4 ± 0.75%, and 26.3 ± 1.37%, respectively, while the apoptotic rate of the control group is 3.77 ± 0.49%. Furthermore, western blot results showed that C-PARP1, C-Caspase-3, and Bax are increased in a dose-dependent manner, while Bcl-2 decreased in a dose-dependent manner (Fig. 3d). Thus, the above data collected suggested that β-Thujaplicin induced apoptosis in HepG2 cells through a mitochondrial-dependent intrinsic pathway.

### β-Thujaplicin induced cell cycle arrest at the S phase in the HepG2 cell line

To assess whether β-Thujaplicin resulted in cell viability decrease involved in cell cycle arrest, the cell cycle was detected. As shown in Fig. 3e, β-Thujaplicin increased the cell number at the S phase in a dose-dependent manner and led to a corresponding decrease in the G2/M phase compared with the DMSO group. Meanwhile, there was only a slight decrease in the G0/G1 phase when compared with high-dose β-Thujaplicin treatment (Fig. 3e, f). Western blot results showed that protein levels of the key checkpoint factors in the S phase, including CDK7, Cyclin D1, and Cyclin A2, were significantly downregulated in cells treated with β-Thujaplicin in a dose-dependent manner, whereas the protein level of P21 was upregulated (Fig. 3h), consistent with the results from real-time PCR (Fig. 3g). All these data verified that the S-phase arrest may be partially accounted to the β-Thujaplicin-reduced viability of HCC cells.

### β-Thujaplicin induced cell apoptosis and the S-phase arrest mediated by activity of p38/ERK MAPK signaling pathway

**Fig. 4:**
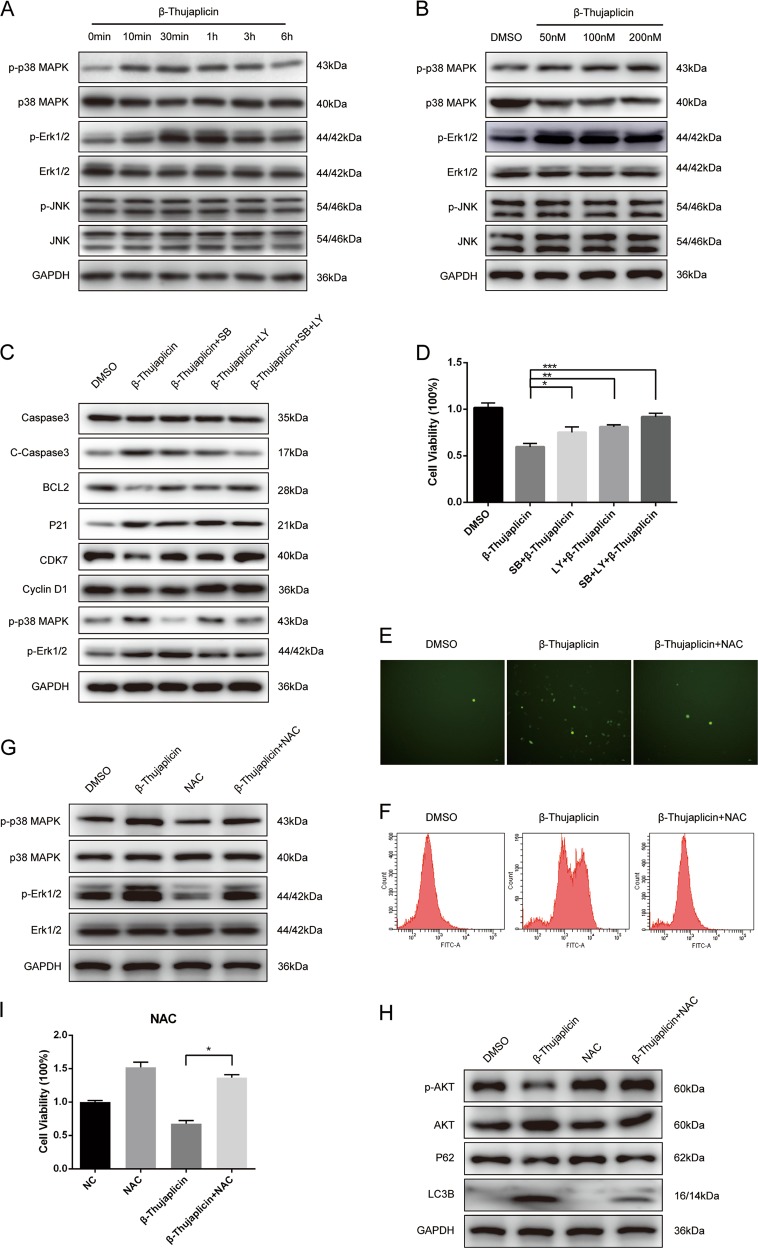
Reactive oxygen species (ROS) accumulation-mediated Akt and p38/ERK MAPK signaling pathway is involved in β-Thujaplicin-induced cancer cell death. **a** The protein levels of p-p38, p38, p-ERK, ERK, p-JNK, and JNK were detected after HepG2 cells were exposed to 100 nM β-Thujaplicin for the indicated time. **b** The protein levels of mitogen-activated protein kinases were determined after HepG2 cells were treated with different concentrations of β-Thujaplicin or 0.2% dimethyl sulfoxide (DMSO) for 1 h. **c**, **d** HepG2 cells were pretreated with or without 0.5 μM SB203580 or 5 nM LY3214996 for 2 h, followed by β-Thujaplicin treatment for an additional 24 h. The expression of c-Caspase-3, Caspase-3, Bcl-2, P21, CDK7, Cyclin D1, p-p38 MAPK, and p-Erk1/2, and cell viability were measured. HepG2 cells were treated with 100 nM β-Thujaplicin with or without 10 mM *N*-acetyl cysteine (NAC) for 24 h, and then intracellular ROS were evaluated by fluorescence microscopy (**e**) and flow cytometry (**f**) using DCFH-DA, and the expression of p-p38, p38, p-ERK, and ERK (**g**), autophagy-related proteins p-Akt, Akt, p62, and LC3B (**h**), and cell viability (**i**) were demonstrated

MAPKs are key signaling proteins that allow eukaryotic cells to respond and interpret to many different extracellular stimuli. We next explored if the effects of β-Thujaplicin on pro-apoptosis and cell cycle arrest in HepG2 cells were mediated by activiting the MAPK signaling pathway. As shown in Fig. 4a, b, β-Thujaplicin significantly increased the phosphorylations of p38 MAP kinase (p38 MAPK) and extracellular signal-regulated kinases (ERK1/2) in a time- and dose-dependent manner, and unexpectedly, the phosphorylation level of c-Jun N-terminal kinases (JNK) was unaffected. To further clarify whether β-Thujaplicin-induced apoptosis and the S-phase arrest depended on p38/ERK MAPK signaling pathways, cells were pretreated with the p38 MAPK inhibitor SB203580 and the ERK inhibitor LY3214996 for 2 h and then treated with β-Thujaplicin for another 24 h. The western blot results showed that β-Thujaplicin induced changes of cell apoptosis and the S-phase arrest protein markers were reversed by SB20358 and LY3214996 (Fig. 4c, d). Furthermore, the MTT assay results indicated that p38 and ERK MAPK inhibitors counteracted the cell viability reducing effect of β-Thujaplicin (Fig. 4i). Overall, these results demonstrated that β-Thujaplicin induced cell apoptosis and the S-phase arrest were p38/ERK MAPK signaling pathway-dependent.

### Intracellular ROS promoted p38/ERK MAPK activation and ACD in β-Thujaplicin-treated cells

ROS plays an important role in cell death induced by chemotherapeutic drugs via regulation of the MAPK signaling pathway. Herein, we tested whether ROS was involved in the cytotoxic effect of β-Thujaplicin. Both fluorescence images and flow cytometric results showed that β-Thujaplicin markedly increased intracellular ROS generation, while antioxidant *N*-acetyl cysteine (NAC) inhibited it (Fig. 4e, f). Moreover, the effects of β-Thujaplicin on cell death were abolished in HepG2 cells treatment with NAC (Fig. 4j). NAC attenuated the increased levels of p-p38 and p-ERK induced by β-Thujaplicin in HepG2 cells, while the expression of p38 and ERK was unchanged (Fig. 4g). We further researched the interrelationship between ROS and β-Thujaplicin-induced autophagy, and found that the upregulated p-Akt and LC3B-II levels that were mediated by β-Thujaplicin were decreased in HepG2 cells treatment with NAC while p62 levels were increased (Fig. 4h). Thus, increased intracellular ROS is proposed as the mechanism which allows β-Thujaplicin to contribute to p38/ERK MAPK pathway-mediated apoptosis and S-phase arrest and Akt pathway-mediated ACD.

### Xenograft tumor growth was inhibited by β-Thujaplicin

**Fig. 5:**
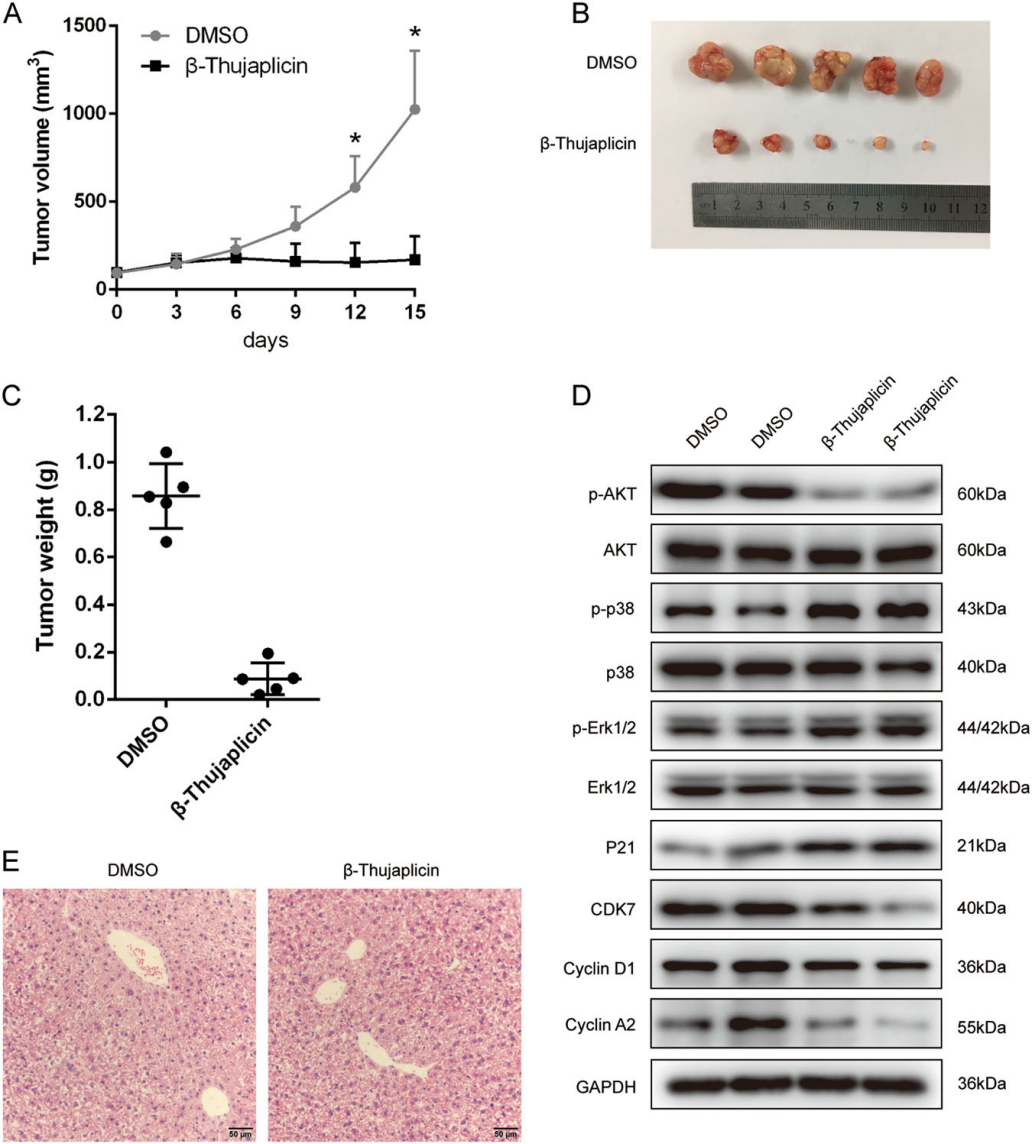
β-Thujaplicin inhibits tumor growth in xenograft mice. **a** Average ± SEM tumor volumes of xenografts in nude mice. Xenograft volumes were monitored at the indicated time using caliper measurements and calculated with the following formula: volume = 0.5 × (length × width^2^). **b** Fifteen days after β-Thujaplicin or dimethyl sulfoxide (DMSO) injection, the mice were euthanized and the xenograft tumors were collected; the image shows the tumors generated by two different treatment groups. **c** Average ± SEM tumor weight was analyzed. **d** The tumor sections were performed by western blot analysis for detection of p-Akt, Akt, p-p38, p38, p-ERK, and ERK. **e** Hematoxylin and eosin staining of liver sections from xenograft mice treated with β-Thujaplicin or DMSO

To further confirm the antitumor effect of β-Thujaplicin in vivo, we constructed xenograft tumor by injection of HepG2 cells to the right flanks of nude mice. When the tumor volume reached about 100 mm^3^, intraperitoneal injection of DMSO or β-Thujaplicin was made, and tumor growth was measured for 15 days. As shown in Fig. 5a, the average tumor volume in the DMSO group was ~1025 ± 272 mm^3^ at the termination of the experiment. By contrast, treatment with β-Thujaplicin significantly reduced the tumor volume, which was 170 ± 108 mm^3^, ~16% of the control group. The excised tumors showed that β-Thujaplicin-treated tumors were much smaller than the control group (Fig. 5b). The average weight of tumors in the β-Thujaplicin group (87 ± 59 mg) was significantly lower than that of the control group (856 ± 121 mg) (Fig. 5c). These results indicated that β-Thujaplicin inhibited xenograft tumor growth in nude mice. In addition, we also found, compared with the control tumors, that the phosphorylation of p38/ERK MAPKs was upregulated and the phosphorylation of Akt was suppressed in xenograft tumors treated with β-Thujaplicin (Fig. 5d). The results of Fig. 5e show that β-Thujaplicin has no significant side effects on normal liver tissues. These results further demonstrated that β-Thujaplicin induced p38/ERK MAPK activation and Akt suppression in HepG2 cells derived from xenograft liver tumor, which may mediated the inhibition effect of β-Thujaplicin on xenograft tumor growth.

## Discussion

Natural bioactive products are a rich source of novel therapeutics derived from plentiful natural substances such as plants, fruits, and mushrooms. Since the 1850s, the structure and function of natural products have been extensively researched. These studies lead to recognition of their anticancer properties. For example, flavonoids, widely distributed in chasteraceae and umbelaceae, were confirmed to be anticancer products^22^. In the present study, β-Thujaplicin, a natural tropolone derivative, was isolated from the heartwood of cupressaceous plants^10^. β-Thujaplicin has shown a remarkable array of biological activities, including antimicrobial, antiviral, anti-inflammatory, and antithrombotic^9–13,23,24^. In addition, some studies have reported that β-Thujaplicin can inhibit proliferation of human breast cancer MCF7 cells^25^, induces apoptosis of human colon cancer HCT-116 cells and teratocarcinoma F9 cells^8,26^, suppresses cancer stemness and oncogenicity in glioma stem U87MG and T98G cells^27^, and may have an ability to overcome EGFR-TKI resistance in lung adenocarcinoma PC9-IR and H1975 cells^28^. But, whether β-Thujaplicin can inhibit liver cancer has not been reported. Our study for the first time validated that β-Thujaplicin restrains liver cancer growth mediated by ACD, apoptosis, and cell cycle arrest.

The Nomenclature Committee on Cell Death used the term “ACD” to define cell death that was accompanied by substantial autophagic vacuolization in the cytoplasm and can be suppressed by inhibiting the autophagy pathway^29^. β-Thujaplicin has shown to suppress the phosphorylation of mTOR and Akt and to increase the LC3-II, thereby inhibiting the melanogenesis in B16F10 mouse melanoma cells^7^. But whether β-Thujaplicin has a function of ACD has not been reported. In our study, we found that β-Thujaplicin could increase autophagosome formation by western blot, confocal microscopy, and transmission electron microscopy analysis. Interestingly, we found that the protein levels of p62 were increased after treatment with 50-nm β-Thujaplicin, but decreased with doses of 100 and 200 nm. This may be due to the relatively weak role of low-dose β-Thujaplicin in promoting the autophagic flux, while a high dose of β-Thujaplicin has a stronger effect on promoting the autophagic flux and degrading more p62, which is a substrate of autophagy. Moreover, we also observed that the total mTOR amount was slightly decreased in HepG2 cells at 50 nm β-Thujaplicin treatment and slightly increased at 100 and 200 nm. This phenomenon was similar to the way RAPA inhibits the mTOR signaling pathway as reported before^30–32^. In specific cell types, such as human lung cancer H460 cells, glioblastoma U87 cells, and nasopharyngeal carcinoma cells, low-dose RAPA can slightly reduce the amount of total mTOR. However, high-dose RAPA inhibits the phosphorylation of mTOR with the total mTOR amount increased. As for the mechanism leading to this strange phenomenon, further research is still needed. Furthermore, we found that blocking the autophagy by CQ and knockdown of ATG5 by siRNA alleviated cell death caused by β-Thujaplicin, while inducing autophagy by RAPA and EBSS aggravated cell death caused by β-Thujaplicin. It was suggested that ACD was involved in β-Thujaplicin-induced HCC cell death. To our knowledge, this is the first report of β-Thujaplicin causing ACD in cancer cell lines.

Previous studies have shown that members of the MAPK family, including p38 MAPK, JNK, and ERK, play an important role in cellular stress response. As originally known, activation of p38 MAPK and JNK is related to apoptosis, whereas activation of ERK is pivotal for cell survival^33,34^. In our study, we have observed an increased phosphorylation of p38 and ERK1/2 after β-Thujaplicin treatment, while there is no change in the phosphorylation level of JNK1/2. Our results are coincident with the recently reported function of p38 MAPK in cancer^35^. However, we also found increased phosphorylation of ERK1/2 in β-Thujaplicin-treated HepG2 cells. Although the ERK signaling is usually important for cell proliferation, controversially, several studies reported that ERK activation can lead to an intrinsic apoptotic pathway resulting in cell death^36,37^. It has been proved that some anticancer drug-induced apoptosis was associated with activation of ERK. The activity of ERK can promote either intrinsic or extrinsic apoptotic pathways and cell cycle arrest. These functions require sustained activation of ERK in specific subcellular organelles and depend on the presence of ROS^38^.

It is well documented that ROS involves autophagy, apoptosis, and cell cycle arrest in many human cancer cell lines^39,40^. Recent studies reported that β-Thujaplicin increased the production of ROS by inactivation of aconitase, thereby causing cytotoxicity in permeabilized yeast cells^41^. In this study, we found that ROS generation is increased in HepG2 cells after exposure to β-Thujaplicin. NAC significantly inhibited the cellular ROS level, decreased p38 MAPK and ERK1/2 activation, and reversed β-Thujaplicin-induced cell death. We also observed that NAC rescued Akt and mTOR activation. These results suggest that β-Thujaplicin-induced ACD, apoptosis, and cell cycle arrest in human HCC cells was closely associated with the increase in intracellular ROS, which may act as upstream factors to regulate the Akt-mTOR and p38/ERK MAPK pathway.

A large number of phytochemicals and other stimuli regulate the Akt-mTOR pathway and the MAPK pathway. Survival and growth of many cancer cells depends on abnormal signal propagation by these pathways, which are intensively involved in crosstalk. There are more than 800 interactive proteins involved in Akt-mTOR-mediated signaling^42^ and more than 2000 interacting proteins related to the MAPK signaling^43^. Due to such numerous interactomes, it is not surprising that the Akt-mTOR pathway and the MAPK pathway affect and contact mutually at different stages of signal transduction, resulting in complicated crosstalk^42,43^. Previous evidence indicates participation of the Akt-mTOR pathway in activation or deactivation of the MAPK cascade, depending on cancer cell types and stimuli. On the other hand, active MAPK can influence the activity of the Akt-mTOR pathway, which is also context dependent^44–46^. In our studies, β-Thujaplicin treatment resulted in decreased activity of the Akt-mTOR pathway and increased activity of the p38/ERK MAPK pathway, simultaneously. Whether there is crosstalk between the two pathways and what are the interactomes under the context of β-Thujaplicin have not been studied. Further experiments are needed to research the relevant mechanisms.

We also confirmed the antitumor effects of β-Thujaplicin by performing an in vivo HepG2 tumor xenograft study in mice. Tumor volume and tumor weight were decreased under β-Thujaplicin treatment, but body weight and the hematological parameters were not significantly affected. Hematoxylin and eosin (H&E) staining shown in Fig. 5e indicates that β-Thujaplicin (intraperitoneal injection, 5 mg/kg body weight, every day) has no significant damage to normal liver tissues. This result is consistent with the study of the pharmacokinetics of β-Thujaplicin in rats^47^. Kenthon Mah et al. administered β-Thujaplicin (30 or 20 mg/kg) to male rats through the jugular vein cannula or by gastric gavage and detected the pharmacokinetic parameters. They found that whether intravenous or oral administration, the half-life of elimination of β-Thujaplicin from blood was no more than 30 min and decreased to undetectable levels within 2 h, with most of it undergoing enterohepatic recycling or excreted by the urinary route and bile. This study also suggests that β-Thujaplicin is relatively nontoxic to laboratory animals.

**Fig. 6:**
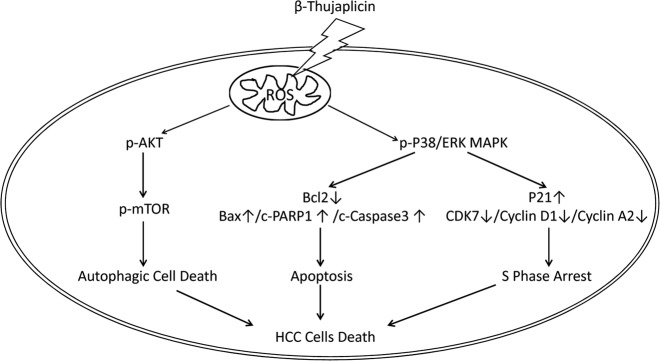
The schematic model of β-Thujaplicin induces autophagic cell death, apoptosis, and cell cycle arrest in hepatocellular carcinoma (HCC) cells. β-Thujaplicin treatment increased the reactive oxygen species (ROS) generation, which subsequently led to inhibition of the Akt-mTOR signaling pathway and activation of p38/ERK MAPK signaling pathway. Consequently, (1) increased expression of LAMP1 and LC3B-II, suggesting that β-Thujaplicin increases autophagosome formation. Furthermore, blocking autophagy can effectively alleviate β-Thujaplicin-induced cell death, while inducing autophagy can aggravate cell death, indicating that β-Thujaplicin has a role in ACD. (2) The increase in cleaved PARP1, cleaved caspase-3, and Bax/Bcl-2 ratio caused activation of the mitochondrial-dependent intrinsic apoptotic pathway. (3) Decreased expression of CDK7, Cyclin D1, and Cyclin A2, and increased expression of p21 caused S-phase arrest. Thus, ROS-mediated p38/ERK MAPKs and the Akt-mTOR signaling pathway was shown to be the underlying mechanism of β-Thujaplicin-induced HCC cell death in this study

In summary, based on our findings, we demonstrated that β-Thujaplicin possesses anti-HCC cancer ability by an in vitro and in vivo study. Mechanistically, our data for the first time showed that β-Thujaplicin triggers ACD, apoptosis, and S-phase arrest to mediate the death of HCC cells. Moreover, we found that β-Thujaplicin induces ACD by inhibiting the Akt-mTOR pathway, and causes apoptosis and S-phase arrest by activating the p38/ERK MAPK pathway. Furthermore, we confirmed that β-Thujaplicin induces the Akt-mTOR pathway inhibition and p38/ERK MAPK pathway activation via accumulation of ROS (Fig. 6). Our study provided insight into the intriguing molecular mechanisms on β-Thujaplicin-induced cell death, which may assist the development of new medicines against HCC.

## Materials and methods

### Reagents and antibodies

β-Thujaplicin were purchased from Sigma-Aldrich (St. Louis, MO, USA). A stock solution of β-Thujaplicin at 100 mM was prepared in DMSO (Sigma, St. Louis, MO, USA) and stored at −20 °C. Dulbecco’s modified Eagle’s medium (DMEM) and phosphate-buffered saline (PBS) were obtained from Basal Media Biotechnology (Shanghai, China). AO/EB staining kit was purchased from Sangon Biotech (Shanghai, China). MTT cell proliferation and cytotoxicity assay kit, BCA protein assay kit, and apoptosis analysis kit were purchased from Beyotime Biotechnology (Suzhou, China). Annexin V-FITC/PI Apoptosis detection kit was purchased from KeyGen Biotech (NanJing, China). Crystal violet solution, CQ, and EBSS were purchased from Sigma-Aldrich (St. Louis, MO, USA) and were used in the concentration indicated before. LY3214996 and RAPA were purchased from Selleck Chemicals (Texas, USA). SB203580 was purchased from MedChemExpress (New Jersey, USA). Ad-GFP-LC3 adenovirus (Hanbio Biotechnology, Shanghai, China) was used to detect autophagosomes. The primary antibodies GAPDH, p-Akt, Akt, p38, p-p38, JNK, p-JNK, ERK, p-ERK, p62, LAMP1, LC3B, cleaved PARP1, cleaved caspase-3, caspase-3, Bax, and Bcl-2 were afforded by Cell Signaling Technology (Beverly, MA, USA).

### Cell culture

Human liver normal cell lines HL-7702 were maintained in RPMI 1640 medium supplemented with 10% fetal bovine serum and 1% penicillin/streptomycin (streptomycin 100 μg/ml, penicillin 100 U/ml) at 37 °C in a humidified atmosphere of 5% CO_2_. Human hepatoma cell lines HepG2, SMMC-7721, and HCCLM3 were maintained in DMEM supplemented with 10% fetal bovine serum and 1% penicillin/streptomycin at 37 ℃ in a humidified atmosphere of 5% CO_2_. All cells were gifted from the Institute of Chinese Academy of Sciences.

### Cell viability assays

For cell viability analysis, MTT Cell Proliferation and Cytotoxicity Assay Kit (Beyotime Biotechnology, Suzhou, China) was used according to the manufacturer’s instructions. Briefly, cells were seeded in 96-well plates (3 × 10^3^/well) and treated with various concentrations of β-Thujaplicin. At the indicated time points, the old medium was replaced with 100 μL of fresh medium containing 10 μl of MTT solution. After 4 h of incubation at 37 °C, formazan crystals were solubilized with 100 μl of formazan dissolution reagent . The absorbance levels for each sample at 570 nm were measured using a microplate reader (Bio-Tek, Winooski, VT, USA). The data were duplicated six times.

### Clone formation assay

Cells were seeded in six-well plates at a density of 500 cells per well. The medium with various concentrations of β-Thujaplicin was refreshed every 3 days for ~12 days when most of the colony contained more than 50 cells. After washing with PBS, the colony was stained with 0.1% crystal violet for 10 min at room temperature. Then the counts of cell colonies were manually scored and the images were recorded by a digital camera.

### Cell cycle analysis

Cells were dispersed on six-well plates at a density of 1 × 10^6^ cells per well and cultured for 24 h. Then the cells were treated with varying concentrations (50, 100, and 200 nM) of β-Thujaplicin for 24 h. After treatment, the cells were collected and washed with ice-cold PBS two times, and then fixed with 70% ethanol for 4 h on ice. Before analysis using flow cytometry, cells were incubated with 100 μl of Rnase (50 μg/ml) and placed at 37 °C for 30 min. Subsequently, 400 μl of propidium iodide was added into the suspension, and cells were stained at 4 °C for 30 min in the dark. Cell cycles were detected using BD FACS Calibur and analysis was conducted with BD CFlow software (BD Biosciences, Mountain View, CA, USA).

### Western blot analysis

Cells were washed two times with ice-cold PBS and lysed in modified RIPA buffer containing protease and phosphatase inhibitors (Roche Diagnostics, Mannheim, Germany) for 30 min. After boiling for 5 min, total protein samples (30 μg/well) were loaded onto 10–12% SDS-polyacrylamide gels and transferred to polyvinylidene fluoride membranes (EMD Millipore, Billerica, MA, USA) at 200 mA for 2 h. For LC3B, 15% gels were used. Membranes were blocked with 5% bovine serum albumin for 1 h before incubation with the corresponding primary antibodies at 4 °C overnight. After washing three times with Tris-buffered saline-Tween buffer, the membranes were then incubated with the horseradish peroxidase-conjugated secondary antibody for 1 h at room temperature. Protein bands were detected using an enhanced chemiluminescence kit (Millipore, Billerica, MA, USA).

### Real-time quantitative PCR analysis

Total RNA was extracted using Trizol reagent (Invitrogen, USA). RNA from each sample of 1 μg was used to synthesize complementary DNA using PrimeScript RT reagent Kit with gDNA Eraser (TaKaRa, Tokyo, Japan) according to the manufacturer’s protocol. For qRT-PCR (real-time quantitative PCR), we used SYBR Premix Ex Taq (TaKaRa, Tokyo, Japan) and the results were normalized to GAPDH. The primer pairs of P21, CDK7, Cyclin D1, Cyclin A2, and GAPDH are P21 F 5′-CGATGGAACTTCGACTTTGTCA-3′, R 5′-CACAAGGGTACAAGACAGTG-3′; CDK7 F 5′-TGTATGGTGTAGGTGTGGACA-3′, CDK7 R 5′-TGCAAAGGTATTCCAGGGAAAC-3′; Cyclin D1 F 5′-CAATGACCCCGCACGATTTC-3′, R 5′-CATGGAGGGCGGATTGGAA-3′; Cyclin A2 F 5′-CGCTGGCGGTACTGAAGTC-3′, R 5′-GAGGAACGGTGACATGCTCAT-3′; and GAPDH (F) 5′-GATGCCCCCATGTTCGTCAT-3′, (R) 5′-TCTTCTGGGTGGCAGTGATG-3′.

### Apoptosis detection by Annexin V-FITC/PI

Annexin V-FITC/PI Apoptosis detection kit (KeyGen Biotech, NanJing, China) was used to measure cell apoptosis. Briefly, cells were plated on a six-well plate and treated with varying concentrations (50, 100, and 200 nM) of β-Thujaplicin for 24 h. Both floating and adherent cells were collected and washed twice with cold PBS. Then the cells were resuspended in 500 μl of binding buffer and incubated with 5 μl of Annexin V-FITC and 5 μl of PI for 15 min at room temperature in the dark. Cell apoptosis was detected using BD FACS Calibur (BD Biosciences, Mountain View, CA, USA).

### Apoptosis detection by AO/EB staining

AO/EB staining kit (Sangon Biotech, Shanghai, China) was used to detect cell apoptosis. Briefly, cells were washed twice with PBS and resuspended in 90 μl of PBS buffer. Then 5 μl of AO Staining Solution and 5 μl of EB Staining Solution were added to a 90-μl cell suspension, and incubated for 5 min at room temperature in the dark. Cells were imaged using a fluorescence microscope Nikon Eclipse Ti-E (Nikon, Japan).

### Measurement of intracellular ROS

ROS generation was measured using the Reactive Oxygen Species Assay Kit (Beyotime Biotechnology, Suzhou, China). After incubation in six-well culture plates for 24 h, cells were treated with different concentrations of β-Thujaplicin. Cells were washed twice with PBS. Then cells were cultured with fresh medium containing 10 μM DCFH-DA (Dichlorofluorescein diacetate) for 20 min. After washing three times with PBS, samples were measured using a microplate reader (Bio-Tek, Winooski, VT, USA) and a fluorescence microscope Nikon Eclipse Ti-E(Nikon, Japan).

### Confocal microscopy

To analyze the autophagy flux in each group, Ad-GFP-LC3 adenovirus (Hanbio Biotechnology, Shanghai, China) was used according to the manufacturer’s instructions. Briefly, 50 MOI adenovirus was added to the medium when cells reached 60% confluence. Cells were incubated for 2 h at 37 °C before being transferred to fresh complete medium. After 36 h for Ad-GFP-LC3 adenovirus infection, cells were treated with DMSO or different concentrations of β-Thujaplicin for 24 h. Fluorescence images were captured by Nikon A1R/A1 Confocal microscopy (Nikon, Japan). GFP dots per cell were used for quantification of autophagosomes. Three regions of interest were selected randomly, and five cells were counted in each group.

### Transmission electron microscopy

After exposure to β-Thujaplicin for 24 h, cells were harvested and fixed with 2.5% glutaraldehyde for 2 h at 4 °C. Then, cells were washed three times with PBS and fixed in 1% aqueous osmium at 4 °C for 1 h, dehydrated with increasing concentrations of acetone, and embedded in araldite. The ultrathin sections were produced with a microtome (Leica, Jena, Germany) and stained with 3% aqueous uranyl acetate and lead citrate. The results were observed by a transmission electron microscope (Hitachi H-7000FA, Tokyo, Japan) and demonstrated as the number of autophagosomes per cell. Autophagosomes are defined as a double-layer membrane structure containing cytoplasmic contents (mitochondria, damaged organelles, etc.) waiting to be degraded. Images of five view fields were taken for data analysis.

### siRNA transfection

siRNA targeting ATG5 and siGenome non-targeting siRNA were used for ATG5 knockdown and control, respectively. The set of siRNAs against ATG5–1 (sense 5′-GGGAAGCAGAACCAUACUATT-3′, antisense 5′-UAGUAUGGUUCUGCUUCCCTT-3′), ATG5–2 (sense 5′-GGAUGCAAUUGAAGCUCAUTT-3′, antisense 5′-AUGAGCUUCAAUUGCAUCCTT-3′), and a negative control siRNA (sense 5′-UUCUCCGAACGUGUCACGUTT-3′, antisense 5′-ACGUGACACGUUCGGAGAATT-3′) were obtained from GenePharma (GenePharma, Shanghai, China). Briefly, cells were grown in six-well plates and transiently transfected at 60–70% confluence with 100 pmol siRNA using Lipofectamine™ RNAi MAX Transfection Reagent (Thermofisher, Massachusetts, USA) according to the manufacturer’s instructions. After 36 h of transfection, the cells were treated with DMSO or with β-Thujaplicin for 24 h. Then the cells were harvested for qRT-PCR western blot and MTT analyses.

### Xenograft tumor growth studies

Male BALB/c nude mice (5–6 weeks old) were purchased from Shanghai Jiesijie Model Animal Research Center (Shanghai, China). All mice were housed under a specific pathogen-free facility and maintained according to the guidelines for laboratory animal care. In total, 5.0 × 10^6^ HepG2 cells in 100 μl of PBS were subcutaneously injected into the right flanks of the mice. When the tumor volume reached about 100 mm^3^, the mice were randomized into two groups and intraperitoneally injected with β-Thujaplicin (5 mg/kg body weight, every day) or 5% DMSO separately. Tumor volume was measured every 3 days for 15 days. Then the mice were euthanized and the tumor and liver were isolated. Tumor volumes were calculated by the following formula: volume = 0.5 × (length × width^2^).

### Immunohistochemistry

After the mice were euthanized, the liver was isolated immediately from β-Thujaplicin or DMSO-treated mice, and fixed in 4% paraformaldehyde solution for 48 h. Then the liver was prepared as paraffin-embedded sections, and used for H&E (Sangon Biotech, Shanghai, China) Staining. The images were captured by Nikon Eclipse Ni-E (Nikon, Japan).

### Statistical analysis

All data were repeated at least three times independently. Data were expressed as the mean ± SD. Statistical significance (*P < 0.05, **P < 0.01, and ***P < 0.001) was determined by GraphPad Prism 5 (GraphPad Software, Inc., La Jolla, CA, USA) using Student’s *t* test or one-way analysis of variance.

## Supplementary information

Supplemental Figure S1

Supplemental Figure S2

Supplemental Figure S3

supplementary figure legends
