# Supplementary figures and images for: β-Thujaplicin induces autophagic cell death, apoptosis, and cell cycle arrest through ROS-mediated Akt and p38/ERK MAPK signaling in human hepatocellular carcinoma
Source: Cell Death Dis. 2019 Mar 15;10(4):255. doi: 10.1038/s41419-019-1492-6 (PMC6420571; doi:10.1038/s41419-019-1492-6)

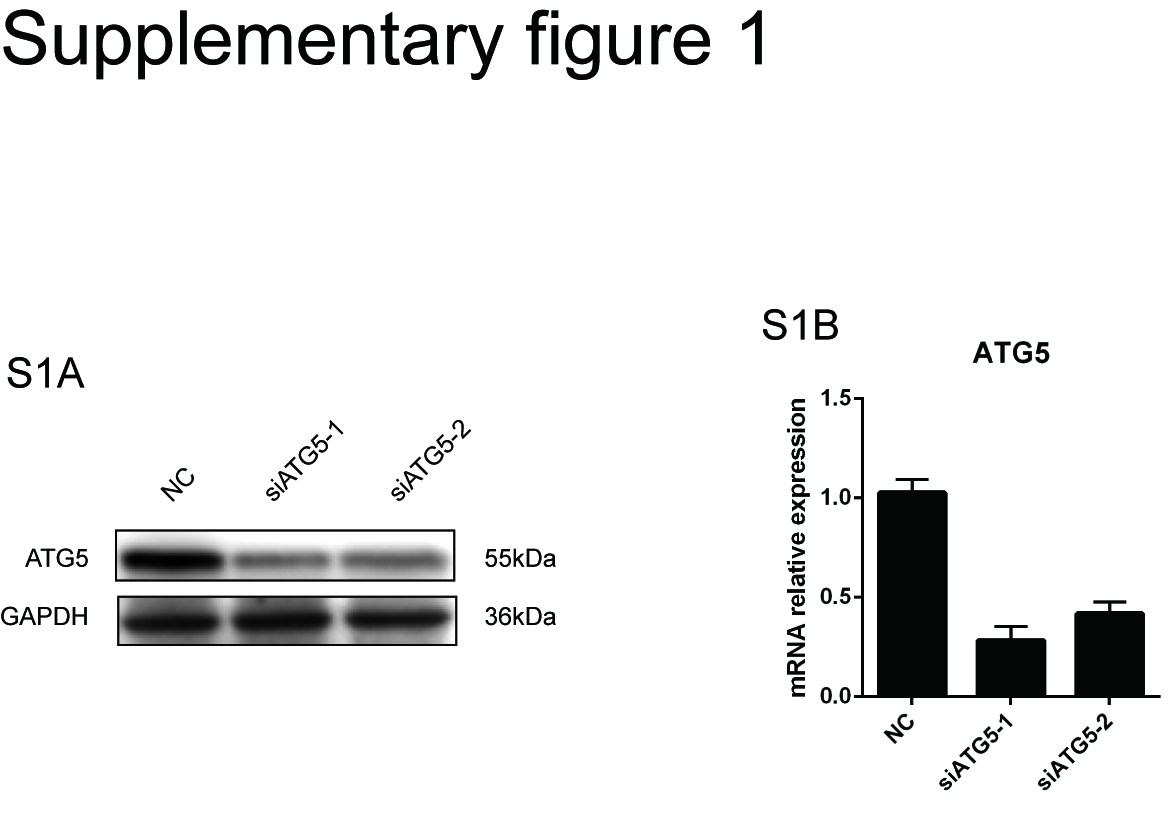

Supplement: Supplementary file 1 — Supplemental Figure S1 [file 41419_2019_1492_MOESM1_ESM.tif]

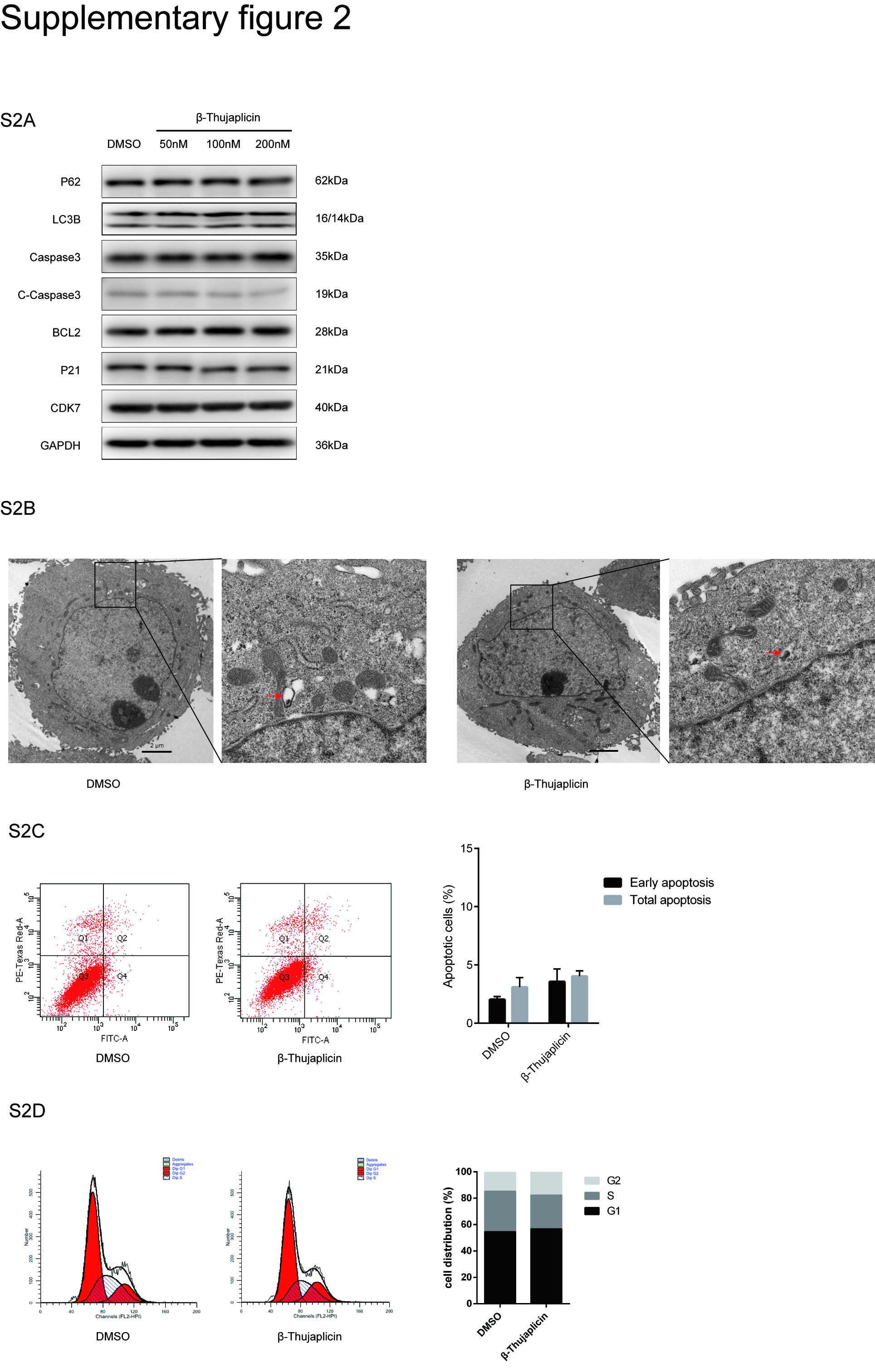

Supplement: Supplementary file 2 — Supplemental Figure S2 [file 41419_2019_1492_MOESM2_ESM.tif]

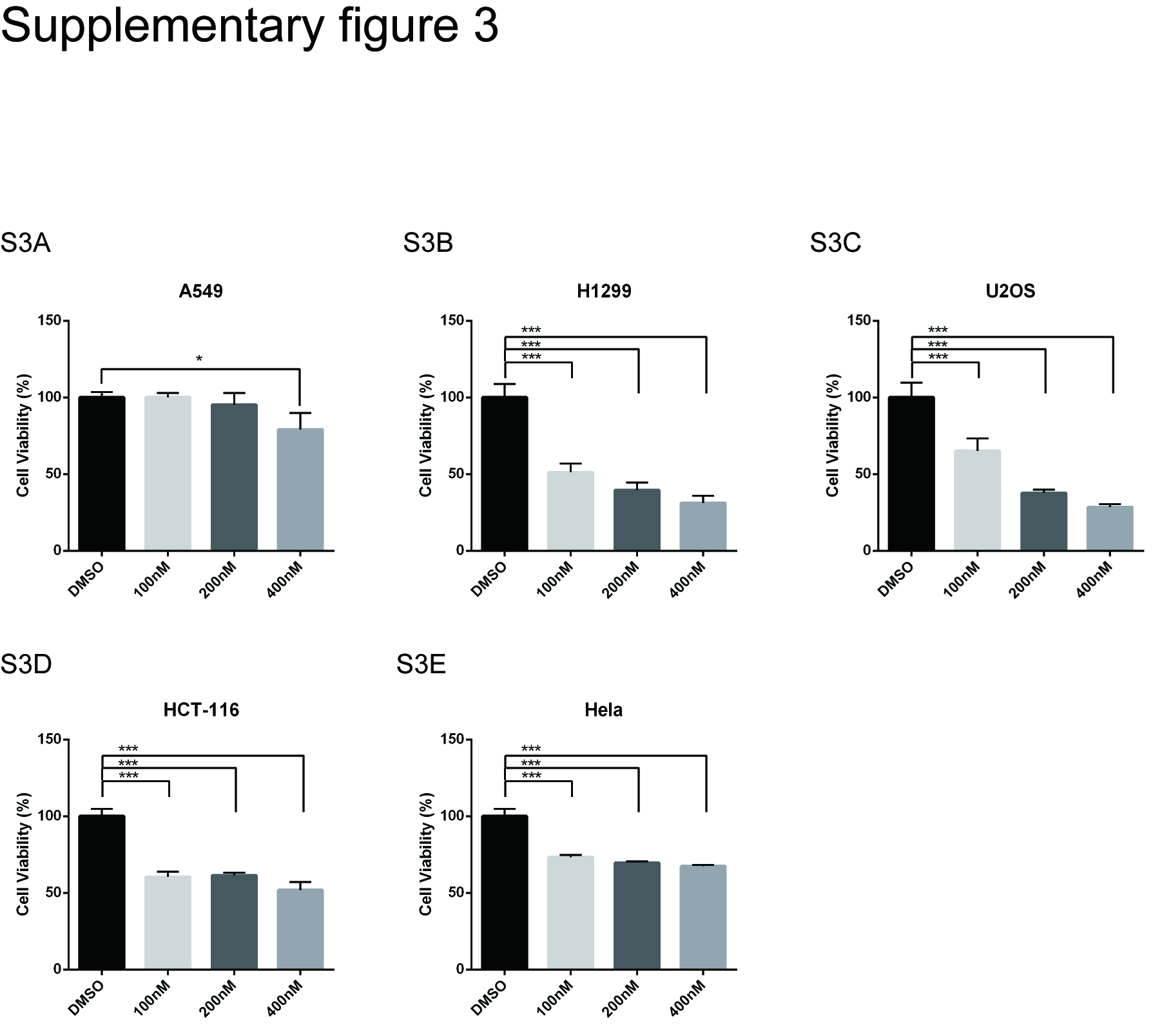

Supplement: Supplementary file 3 — Supplemental Figure S3 [file 41419_2019_1492_MOESM3_ESM.tif]
