# Supplementary material for: β-Thujaplicin induces autophagic cell death, apoptosis, and cell cycle arrest through ROS-mediated Akt and p38/ERK MAPK signaling in human hepatocellular carcinoma
Source: Cell Death Dis. 2019 Mar 15;10(4):255. doi: 10.1038/s41419-019-1492-6 (PMC6420571; doi:10.1038/s41419-019-1492-6)
Supplement: Supplementary file 4 — supplementary figure legends [file 41419_2019_1492_MOESM4_ESM.docx]

**supplementary figure legends**

**Supplemental Figure 1** Protein and mRNA levels of ATG5 in HepG2 cells transfected with specific ATG5 siRNAs or scramble siRNA. All experiments were performed in triplicate. *P<0.05.

**Supplemental Figure 2** (a) The levels of autophagy, apoptosis and cell cycle related proteins were detected after HL-7702 cells exposed to 0.2% DMSO or 50, 100, 200 nM β-Thujaplicin for 24 h. (b) Representative TEM pictures of autophagosomes (arrow) in 100 nM β-Thujaplicin or 0.2% DMSO treated HL-7702 cells. (c and d) HL-7702 cells were treated with 100 nM β-Thujaplicin or 0.2% DMSO for 24 h, and then the ratio of apoptosis and cell cycle distribution were measured by flow cytometry.

**Supplemental Figure 3**  (c to e) Cancer cell line A549 (a), H1299 (b), U2OS (c), HCT116 (d) and Hela (e) cells were treated with different concentrations of β-Thujaplicin or 0.2% DMSO for 24 h, and MTT assay was used to measure cell viability. *P<0.05, **P<0.01, ***P<0.001.
